# Hot Days in Early Pregnancy: A Potential Risk Factor for Congenital Heart Defects

**DOI:** 10.1289/ehp.125-A25

**Published:** 2017-01-01

**Authors:** Lindsey Konkel

**Affiliations:** Lindsey Konkel is a New Jersey–based journalist who reports on science, health, and the environment.

If the frequency and intensity of heat waves continue to increase in certain parts of the world as predicted,^1^ more people are likely to be exposed to prolonged periods of hot days. A new study in *EHP* explores women’s exposure to high heat during early pregnancy as a possible risk factor for congenital heart defects in their babies.^2^

**Figure:**
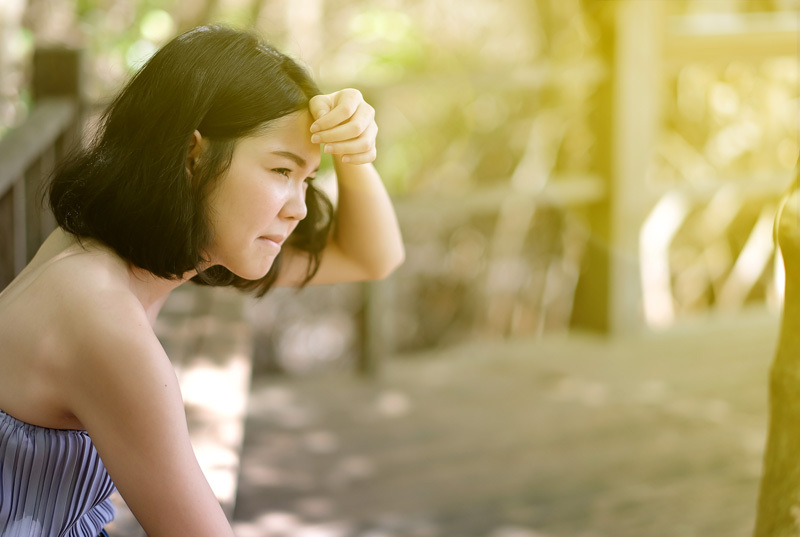
Only a few studies have investigated mothers’ exposure to hot days in the first weeks of pregnancy as a risk factor for congenital heart defects in their babies. © Chayathorn Lertpanyaroj/Shutterstock

The fetal heart begins to form in the earliest weeks of gestation.^3^ About 8 in every 1,000 infants are born with some form of congenital heart defect, making these the most common type of birth defects.^4^ Three-quarters of congenital heart defects are noncritical, meaning the infant likely won’t need surgery or other procedures in the first year of life. Critical congenital heart defects, although less common, are more severe, with many requiring treatment soon after birth.^5^

It is not clear what causes most congenital heart defects, although previous research has suggested that factors such as maternal smoking and diabetes may elevate risk.^6^ Maternal fever during the first trimester has been associated with congenital heart defects in some human studies,^7^ ^,^ ^8^ although earlier studies on maternal exposure to hot days did not find strong associations.^9^ ^,^ ^10^

In the current study, lead author Nathalie Auger, an epidemiologist at the University of Montreal Hospital Research Centre, and colleagues analyzed records for infants born in Quebec between 1988 and 2012. They calculated each baby’s conception date based on gestational age at delivery, and identified more than 700,000 babies conceived between the months of April and September.

Next, they compared the prevalence of seven critical heart defects and eight noncritical heart defects in these infants against the number of hot days to which mothers would have been exposed between weeks 2 and 8 of pregnancy. The researchers counted days in which maximum outdoor temperatures reached 30°C (86°F) or above.

Roughly 980 babies per 100,000 exposed to 10 or more hot days in weeks 2–8 were born with a congenital heart defect, compared with roughly 879 babies per 100,000 with zero days of exposure. Exposure to at least 15 hot days during weeks 2–8 was associated with a 37% higher prevalence of atrial septal defects compared with zero exposure.^1^

“We would expect that risk would be small, because we would not expect temperature to have a strong effect,” says Shao Lin, an epidemiologist and professor at the University at Albany, State University of New York. She explains that if maternal heat exposure were a major risk factor for heart defects the same as, say, smoking is for lung cancer, we would already know it. She says temperature is more likely just one of several risk factors, and any adverse birth effect could be due to joint or mediating effects between heat and maternal chronic diseases, use of certain medications, or activity patterns. Lin was not involved in the study.

Although the researchers based their findings on measures of outdoor temperature, they lacked information on how much time the women actually spent outside on hot days. They also didn’t know what temperatures the mothers were exposed to inside their homes or workplaces. The impact of outdoor heat could be mitigated by air conditioning or worsened in urban areas that retain high heat.^2^

It is still not clear how outside heat might affect heart formation, although Auger and colleagues point to studies that have suggested high temperatures could alter cell physiology.^11^ Previous research has also found associations between air pollution and specific congenital heart defects.^12^ But it is unknown how other environmental factors related to heat waves—such as an increase in levels of certain air pollutants—might interact with the effects of temperature or whether those other factors could even be responsible for the association, says Martine Vrijheid, an epidemiologist at the Barcelona Institute for Global Health. Vrijheid was not involved in the study.

Vrijheid says future studies should explore factors such as air pollution and indoor temperature in relation to hot weather, and they should be conducted in other populations. “It’s well known that the health effects of heat waves depend on the normal temperatures in a given location,” she explains—for instance, the temperatures that constitute a heat wave are lower in Quebec than in, say, Florida.

Lin adds that studies also should control for time-varying factors such as long-term trends and vacations, and separate summer months from spring and fall months. “Heat’s effects could be quite different by season,” she says.
